# PAGE-based transfer learning from single-cell to bulk sequencing enhances model generalization for sepsis diagnosis

**DOI:** 10.1093/bib/bbae661

**Published:** 2024-11-22

**Authors:** Nana Jin, Chuanchuan Nan, Wanyang Li, Peijing Lin, Yu Xin, Jun Wang, Yuelong Chen, Yuanhao Wang, Kaijiang Yu, Changsong Wang, Chunbo Chen, Qingshan Geng, Lixin Cheng

**Affiliations:** Guangdong Provincial Clinical Research Center for Geriatrics; Shenzhen Clinical Research Center for Geriatrics, Shenzhen People’s Hospital, the Second Clinical Medical College of Jinan University, the First Affiliated Hospital of Southern University of Science and Technology, 1017 Dongmen Rd N, Luohu District, Shenzhen 518020, China; Post-doctoral Scientific Research Station of Basic Medicine, Jinan University, 601 Huangpu Blvd W, Tianhe District, Guangzhou 510632, China; Department of Critical Care Medicine, Shenzhen People’s Hospital, the Second Clinical Medical College of Jinan University, the First Affiliated Hospital of Southern University of Science and Technology, 1017 Dongmen Rd N, Luohu District, Shenzhen 518020, China; Guangdong Provincial Clinical Research Center for Geriatrics; Shenzhen Clinical Research Center for Geriatrics, Shenzhen People’s Hospital, the Second Clinical Medical College of Jinan University, the First Affiliated Hospital of Southern University of Science and Technology, 1017 Dongmen Rd N, Luohu District, Shenzhen 518020, China; Guangdong Provincial Clinical Research Center for Geriatrics; Shenzhen Clinical Research Center for Geriatrics, Shenzhen People’s Hospital, the Second Clinical Medical College of Jinan University, the First Affiliated Hospital of Southern University of Science and Technology, 1017 Dongmen Rd N, Luohu District, Shenzhen 518020, China; Department of Critical Care Medicine, the First Affiliated Hospital of Harbin Medical University, 23 Youzheng Street, Nangang District, Harbin, Heilongjiang 150001, China; Bioinformatics Centre, Department of Biology, University of Copenhagen, Ole Maaløes Vej 5, 2200 København, Denmark; School of Life Sciences, The Chinese University of Hong Kong, Shatin, Hong Kong SAR, 999077, China; Guangdong Provincial Clinical Research Center for Geriatrics; Shenzhen Clinical Research Center for Geriatrics, Shenzhen People’s Hospital, the Second Clinical Medical College of Jinan University, the First Affiliated Hospital of Southern University of Science and Technology, 1017 Dongmen Rd N, Luohu District, Shenzhen 518020, China; Department of Critical Care Medicine, the First Affiliated Hospital of Harbin Medical University, 23 Youzheng Street, Nangang District, Harbin, Heilongjiang 150001, China; Department of Critical Care Medicine, the First Affiliated Hospital of Harbin Medical University, 23 Youzheng Street, Nangang District, Harbin, Heilongjiang 150001, China; Department of Critical Care Medicine, Shenzhen People’s Hospital, the Second Clinical Medical College of Jinan University, the First Affiliated Hospital of Southern University of Science and Technology, 1017 Dongmen Rd N, Luohu District, Shenzhen 518020, China; Guangdong Provincial Clinical Research Center for Geriatrics; Shenzhen Clinical Research Center for Geriatrics, Shenzhen People’s Hospital, the Second Clinical Medical College of Jinan University, the First Affiliated Hospital of Southern University of Science and Technology, 1017 Dongmen Rd N, Luohu District, Shenzhen 518020, China; Guangdong Provincial Clinical Research Center for Geriatrics; Shenzhen Clinical Research Center for Geriatrics, Shenzhen People’s Hospital, the Second Clinical Medical College of Jinan University, the First Affiliated Hospital of Southern University of Science and Technology, 1017 Dongmen Rd N, Luohu District, Shenzhen 518020, China; Department of Critical Care Medicine, Shenzhen People’s Hospital, the Second Clinical Medical College of Jinan University, the First Affiliated Hospital of Southern University of Science and Technology, 1017 Dongmen Rd N, Luohu District, Shenzhen 518020, China; Health Data Science Center, Shenzhen People's Hospital, 1017 Dongmen Rd N, Luohu District, Shenzhen 518020, China

**Keywords:** transfer learning, single-cell transcriptome, sepsis diagnosis, gene expression

## Abstract

Sepsis, caused by infections, sparks a dangerous bodily response. The transcriptional expression patterns of host responses aid in the diagnosis of sepsis, but the challenge lies in their limited generalization capabilities. To facilitate sepsis diagnosis, we present an updated version of single-cell Pair-wise Analysis of Gene Expression (scPAGE) using transfer learning method, scPAGE2, dedicated to data fusion between single-cell and bulk transcriptome. Compared to scPAGE, the upgrade to scPAGE2 featured ameliorated Differentially Expressed Gene Pairs (DEPs) for pretraining a model in single-cell transcriptome and retrained it using bulk transcriptome data to construct a sepsis diagnostic model, which effectively transferred cell-layer information from single-cell to bulk transcriptome. Seven datasets across three transcriptome platforms and fluorescence-activated cell sorting (FACS) were used for performance validation. The model involved four DEPs, showing robust performance across next-generation sequencing and microarray platforms, surpassing state-of-the-art models with an average AUROC of 0.947 and an average AUPRC of 0.987. Analysis of scRNA-seq data reveals higher cell proportions with JAM3-PIK3AP1 expression in sepsis monocytes, decreased ARG1-CCR7 in B and T cells. Elevated IRF6-HP in sepsis monocytes confirmed by both scRNA-seq and an independent cohort using FACS. Both the superior performance of the model and the *in vitro* validation of IRF6-HP in monocytes emphasize that scPAGE2 is effective and robust in the construction of sepsis diagnostic model. We additionally applied scPAGE2 to acute myeloid leukemia and demonstrated its superior classification performance. Overall, we provided a strategy to improve the generalizability of classification model that can be adapted to a broad range of clinical prediction scenarios.

## Introduction

Sepsis arises when the body’s immune response to an infection triggers systemic inflammation, causing dysfunction or failure of multiple organs. It is a life-threatening medical emergency that resulted in 48.9 million cases and 11 million deaths worldwide in 2017, accounting for almost 20% of all global deaths [1]. Prompt recognition, effective management, and early initiation of appropriate treatment are crucial in improving outcomes for sepsis patients. Researches in understanding the pathophysiology of sepsis, early warning systems, and evidence-based guidelines have resulted in improved sepsis care [2]. However, the early diagnosis of sepsis remains a significant healthcare challenge that needs to be addressed.

Benefiting from the rapid development of sequencing technology, several studies have utilized transcriptome profiles to develop diagnostic models that can distinguish between patients with sepsis and those with non-infectious inflammation [3–10]. For example, Scicluna *et al.* identified a sNIP score using the gene expression of three genes to stratify patients with abdominal sepsis [5]. McHugh et al. developed a four-gene classifier combining expression of CEACAM4, LAMP1, PLA2G7, and PLAC8 genes in managing ICU patients with systemic inflammation [11]. However, the models developed in these studies were constructed using multiple small sepsis cohorts. This approach presents a challenge as the reliability of measurements for the expression of individual genes can be affected by factors such as differences in sample collection, processing methods, and sequencing platforms across the diverse data sources.

Instead of relying on individual genes, strategies like Pair-wise Analysis of Gene Expression (PAGE) employ pair-wise comparisons of gene expression abundance within samples to construct models [12–17]. This approach provides a more robust foundation for constructing models by sophisticatedly avoiding comparisons across samples, aiming to mitigate the confounding influence introduced by batch effects. For instance, Zheng *et al*. discovered a signature consisting of 14 pairs of long non-coding RNAs (lncRNAs), which aids in the diagnosis of septic patients at an early stage, when clinical manifestations may not be pronounced [4]. The previous methods we developed, bvnGPS and GPGPS [14, 15], incorporated aggregated data from different platforms for training, demonstrating superior generalization across datasets and platforms. Therefore, using PAGE can overcome the limitations imposed by diverse transcriptome platforms.

Besides traditional bulk RNA-seq data, single-cell RNA (scRNA) sequencing data can also benefit from the use of PAGE and can be employed for patient stratification [13]. scRNA sequencing (scRNA-seq) provides higher resolution to reveal specific gene expression profiles in individual cells, uncovering cell-to-cell variation and identifying rare cell types or subpopulations that may be missed in bulk RNA sequencing [18–20]. Given the constraints of a limited sample size in bulk RNA-seq data within a single study, PAGE can assist in constructing models using scRNA data and then effectively apply and generalize the findings to bulk data. In this approach, PAGE’s advantage lies in that it does not compare the expression levels between samples, making it robust for handling data from different platforms [21]. Furthermore, it utilizes the relative expression between genes, thereby being less affected by the noise in scRNA-seq data. Finally, the relative expression between genes helps alleviate the dropout issue in single-cell technology. Wang et al. introduced a technique called single-cell pairwise analysis of gene expression (scPAGE) to assist in identifying gene expression signatures for classifying cells from patients with and without sepsis, following by applied to bulk RNA-seq datasets, demonstrating effective classification performance [13].

Despite demonstrating its generalizability to bulk data, scPAGE solely relies on scRNA-seq data information, lacks the consideration of information from bulk data during modeling. In this study, we developed scPAGE2, a novel approach that leverages information from both bulk and scRNA-seq datasets using transfer learning strategy [22]. We first pre-trained the single-cell Gene Pair Signatures (scGPS) model with scPAGE using an enhanced strategy for calculating the relative expression within a gene pair. Subsequently, we retrained scGPS using bulk transcriptome data to transfer cell-layer information from single-cell to bulk transcriptome. The refined model, referred to as scGPS2, was constructed using four Differentially Expressed Gene Pairs (DEPs) and demonstrated strong and consistent performance across both next-generation sequencing (NGS and microarray platforms, as well as against state-of-the-art classification models. Our analysis of the single-cell dataset pinpointed that ARG1-CCR7 was a prominent indicator for B cells and T cells in sepsis, while JAM3-PIK3AP1 and IRF6-HP were identified as notable marker for monocytes in sepsis. The high expression of HP in monocyte of sepsis was further validated using fluorescence-activated cell sorting (FACS).

## Materials and methods

### Data collection

The Single-cell 3’ mRNA sequencing data from four sepsis patients and six healthy controls (P02H, P04H, P06F, P07H, P08H, and P09H) were obtained from the Broad Institute Single Cell portal (https://singlecell.broadinstitute.org/single_cell), study number SCP548 [23]. This dataset includes a total of 13,254 cells after filtering out cells with fewer than 500 detected genes. The cells are annotated with their respective cell types, which include B cell, dendritic cell (DC), monocyte, natural killer cell (NK), and T cell.

To retrain scGPS2, the gene expression data of human peripheral blood mononuclear cells (PBMCs) from 221 individuals were downloaded from the GEO database (Accession No. GSE189400) [24], serving as the fine-tuning dataset. The dataset consists of 129 sepsis samples and 92 normal samples. In addition, seven independent external test sets, comprising a total of 1184 samples, were utilized for evaluation: GSE185263, GSE26378, GSE26440, GSE57065, GSE66099, GSE69528, and GSE95233 [25–30]. The training dataset constitutes 15.7% of the total, with the test datasets accounting for the remaining 84.3%. The characteristics of patients, sample size and platform information for each dataset are described in Table 1. For NGS platforms (GSE185263 and GSE189400), gene expression was measured using transcript per million (TPM). For microarray platforms, quantile normalization (GSE69528 and GSE66099), and log2 normalization (GSE57065, GSE95233, GSE26378, and GSE26440) were used to normalize the gene expression.

**Table 1:** Characteristics of patients in the RNA-seq datasets.

Dataset	Technology	Platform	Human immune-related gene count	References	Total number		No. of samples	Age	Gender		Mortality (In hospital/28-day)
									Male	Female	
GSE185263	RNA-seq	GPL16791	958	[25]	392	Sepsis	348	57.4 ± 1.05	202 (58.0%)	146 (42.0%)	52 (14.9%)
						Normal	44	46.4 ± 2.43	18 (40.9%)	26 (59.1%)	-
GSE189400	RNA-seq	GPL24676	925	[24]	221	Sepsis	129	64.7 ± 1.37	81 (62.8%)	47 (36.4%)	38 (29.5%)
						Normal	92	63.3 ± 1.60	53 (57.6%)	39 (42.4%)	32 (34.8%)
GSE69528	RNA-seq	GPL10558	729	[30]	82	Sepsis	54	-	-	-	-
						Normal	28	-	-	-	-
GSE26378	Microarray	GPL570	911	[26]	103	Sepsis	82	3.71 ± 0.37	-	-	-
						Normal	21	3.87 ± 0.65	-	-	-
GSE26440	Microarray	GPL570	911	[28]	130	Sepsis	98	3.36 ± 0.31	-	-	-
						Normal	32	2.41 ± 0.45	-	-	-
GSE57065	Microarray	GPL570	911	[27]	107	Sepsis	82	62.7 ± 1.71	55 (67.1%)	27 (32.9%)	-
						Normal	25	44.5 ± 2.23	5 (20%)	20 (80%)	-
GSE66099	Microarray	GPL570	911	[29]	246	Sepsis	199	-	-	-	-
						Normal	47	-	-	-	-
GSE95233	Microarray	GPL570	911	[27]	124	Sepsis	102	62.2 ± 1.48	62 (60.8%)	36 (35.3%)	-
						Normal	22	57.8 ± 1.35	11 (50%)	11 (50%)	-

**Note:** The mean value ± standard error is presented for numerical variables.

Single-cell datasets of acute myeloid leukemia (AML) and normal mouse (*Mus musculus*) bone marrow were collected from the GEO database (Accession No. GSE128423), as described in previous work [13]. GSE74690, which includes 19 leukemia samples and 30 normal samples, was used to fine-tune scGPS2 for AML. Five independent external datasets—GSE105049 (N = 12), GSE121122 (N = 36), GSE132948 (N = 18), GSE78691 (N = 11), and GSE84988 (N = 12)—were utilized to evaluate the model. The expression levels were converted to TPM and subsequently log2 transformed.

### Overview of scPAGE2

**Figure 1:**
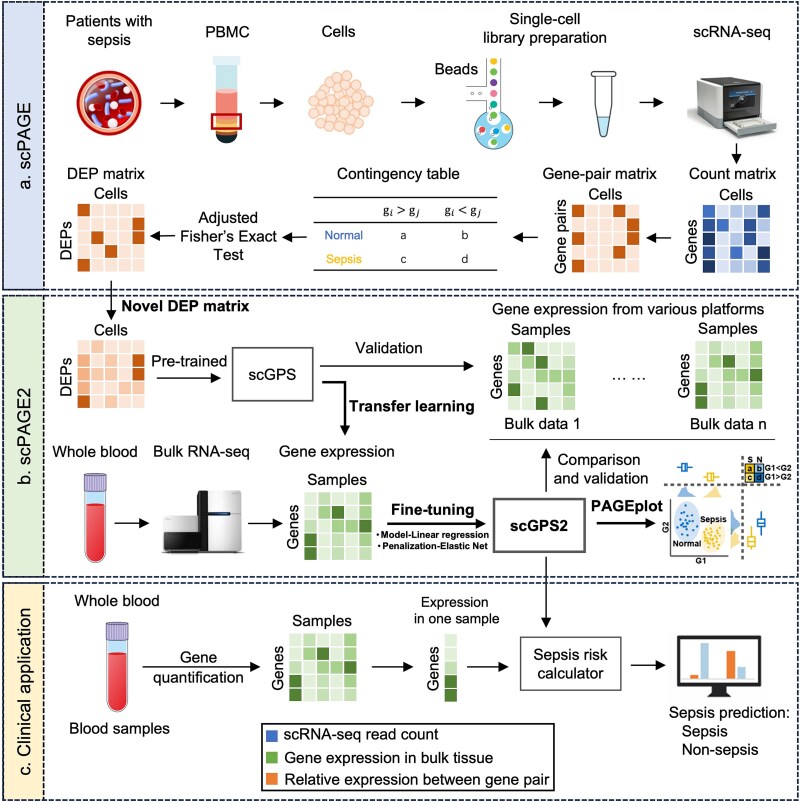
Workflow depicting the study’s progression. (a) Initial scPAGE algorithm illustrating cell isolation from sepsis patient PBMC samples, followed by single-cell library preparation and subsequent conversion of count matrix to gene-pair matrix for significant DEP identification. (b) Enhanced scPAGE2 algorithm showcasing DEP refinement and re-training of the scGPS model using the EN, with subsequent evaluation of scGPS2. (c) Clinical application of scPAGE2 involving individualized sepsis risk prediction for each blood sample.

Figure 1 illustrates the overview of scPAGE2. Based on our previous scPAGE method, we utilized the PAGE algorithm to identify 22 pairs of DEPs for sepsis patient classification. These DEPs are discrete, which may result in information loss and difficulties in handling missing values when constructing models using discrete variables. This could lead to the loss of key information and the introduction of bias or uncertainty, thereby affecting accuracy. To address these challenges, we instead improved the model by converting DEPs to continuous variables in the pre-training phase. Then, we implemented scGPS model, which was originally developed from single-cell data and exhibited strong performance in bulk RNA-seq data using scPAGE. To amalgamate valuable insights from both scRNA-seq and bulk RNA-seq data, we conducted transfer learning on bulk RNA-seq data, which involved retraining the scGPS model using elastic net (EN), resulting in an improved model, scGPS2, with better classification performance. The comparison between scPAGE and scPAGE2 was illustrated in Table 2. The scGPS2 model leverages multi-sourced gene expression data from patient blood samples to predict the personalized risk of sepsis, facilitating early detection and treatment decisions for sepsis.

**Table 2:** Comparison between scPAGE and scPAGE2.

		scPAGE	scPAGE2
Data	Training	SCP548	SCP548
	Retraining	-	GSE189400
	External validation	Five microarray datasets: GSE13015, GSE28750, GSE57065, GSE66099, GSE69528	Seven microarray and RNA-seq datasets: GSE185263, GSE26378, GSE26440, GSE57065, GSE66099, GSE69528, GSE95233
	Platforms for validation	Microarray	Microarray, RNA-seq
Methods	Model construction	Train using a single-cell dataset	Pre-train on a single-cell dataset and fine-tune using a bulk RNA-seq dataset
	Determination methods of DEPs	Discrete values (Equation 1)	Continuous values (Equation 2)
	Model complexity	Linear model (22 DEPs)	Linear model (4 DEPs)
Results	Pair signatures	ARG1-CCR7, ARG1-CLEC4C, ARG1-CXCR3, ARG1-IL18R1, ARG1-PROCR, CAV1-JAM3, DUSP1-TLR9, IRF6-HP, JAM3-PIK3AP1, LGMN-ARG1, MUC1-HP, PGLYRP2-TLR9, PPARG-CXCR3, PPARG-IL18R1, PPARG-PROCR, TLR2-TLR9, TLR9-CLEC4D, TLR9-EGR1, TLR9-GAB1, TLR9-HP, TLR9-MAFB, and TLR9-S100A12	ARG1-CCR7, IRF6-HP, JAM3-PIK3AP1, and TLR9-CLEC4D

### Single-cell pair-wise analysis of gene expression

To classify sepsis patients, our previous study proposed a PAGE-based method called scPAGE, which can effectively transfer models from single-cell data to bulk data.

First, 1040 human immune genes collected from InnateDB (https://www.innatedb.com/annotatedGenes.do?type=innatedb) [31] was used to filter the expression profile $E$. Then, a gene-pair matrix $M$ is defined from the expression profile $E$, representing the relative expression levels of $L$ gene pairs in $K$ cells. For the gene pair $(l)$ consisting of genes $i$ and $j$ in the cell $k$,


(1) \begin{equation*} {m}_{lk}=\left\{\begin{array}{@{}ll}1,& if\ {e}_{ik}>{e}_{jk}\\{}-1,& if\ {e}_{ik}\le{e}_{jk}\end{array}.\right. \end{equation*}

For each gene pair $(l)$, we calculated the significance of the reverse expression pattern exhibited by that gene pair in sepsis and normal samples using Fisher’s exact test. The contingency table was shown in the top panel of Fig. 1. The significant DEPs were determined by the adjusted P-value ≤0.05 after Bonferroni correction. Then, based on the sequential forward selection (SFS) method, the optimal panel of DEPs was selected to construct scGPS, which can effectively separate sepsis and normal cells. Finally, the scGPS was transferred and applied to five independent validation sets to optimize and evaluate the model.

### Enhanced approach for identifying DEPs using continuous variables

Using discrete variables in model construction has two drawbacks. Firstly, using discrete variables for gene pairs in modeling only captures the relative expression level, disregarding the degree of relative expression. Consequently, this process may lead to the loss of crucial information, ultimately impacting the accuracy of the model. Secondly, handling missing values in modeling discrete variables can pose substantial difficulties. One common approach to addressing missing values in discrete variables is by utilizing a unique representation; however, this approach may introduce bias and uncertainty.

To address the aforementioned shortcomings, we constructed scGPS2 based on an enhanced approach for identifying DEPs. Rather than employing Equation (1), the following formulation was used:


(2) \begin{equation*} {m}_{lk}={e}_{ik}-{e}_{jk}, \end{equation*}

where ${m}_{lk}$ represented the degree of relative expression level for gene pair $(l)$ in cell $k$. Here, $i$ and $j$ represented the two genes in the gene pair $(l)$ in cell $k$.

### Construction of scGPS2 using transfer learning

Previously, twenty-two DEPs were identified in scGPS (as shown in Supplementary Table S1) [13]. Due to the limited sample size of the bulk data, we employed transfer learning, a machine learning technique that involves adapting or fine-tuning a model originally trained for one data source to perform a related but distinct data source. This approach allowed us to utilize the knowledge and features acquired from scRNA-seq data and apply them effectively to the bulk RNA-seq data, enhancing our model’s performance. As a result, we integrated the 22 DEPs by utilizing their relative expression values (as defined in Equation 2). We employed the EN algorithm to train the prediction model on the GSE189400 dataset, yielding the improved model known as scGPS2.

The cost function of the EN regression algorithm combines the regularization techniques of both Lasso regression and Ridge regression, using two parameters, λ and ρ, to control the magnitude of the penalty terms (see Equation 3).


(3) \begin{equation*} W={}_w{}^{argmin}\left(\sum_{i=1}^N{\left({y}_i-{w}^T{x}_i\right)}^2+\lambda \rho{\left\Vert w\right\Vert}_1+\frac{\lambda \left(1-\rho \right)}{2}{\left\Vert w\right\Vert}_2^2\right), \end{equation*}

where $\lambda \rho{\left\Vert w\right\Vert}_1+\frac{\lambda \left(1-\rho \right)}{2}{\left\Vert w\right\Vert}_2^2$represents a convex linear combination of the penalty functions for Ridge regression (L2 regularization) and Lasso regression (L1 regularization). When ρ = 0, EN regression reduces to Ridge regression, and when ρ = 1, EN regression becomes equivalent to Lasso regression. Therefore, EN regression combines the advantages of both Lasso and Ridge regression. It achieves the goal of feature selection for important features while excluding those that have minimal impact on the dependent variable.

By utilizing the built-in ElasticNet function from the Python scikit-learn library (v0.22), we conducted multiple parameter tuning iterations to select the most suitable values (alpha = 0.23, l1_ratio = 0.32) (where alpha corresponds to λ and l1_ratio corresponds to ρ). Ultimately, this process led to the identification of five DEPs as feature factors, namely ARG1_CCR7, IRF6_HP, JAM3_PIK3AP1, TLR9_CLEC4D, and TLR9_HP. In the process of comparing the model’s classification performance with and without TLR9_HP, we manually excluded this DEP from the model. The finalized risk score for sepsis was determined as follows:


(4) \begin{equation*} Y=\sum_{i=1}^N\left({Coefficient}_i\ast Relative\_{expression}_i\right) \end{equation*}

where $Y$ represented the risk score for sepsis, $N$ represented the number of DEPs, $Coefficient$ of ARG1_CCR7, IRF6_HP, JAM3_PIK3AP1, and TLR9_CLEC4D corresponded to 0.01439, 0.0129, −0.01868, and − 0.03649, respectively. $Relative\_ expression$ of these DEPs corresponded to the values for $ARG1- CCR7$, $IRF6- HP$, $JAM3- PIK3 AP1$, and $TLR9- CLEC4D$.

### DEP visualization

To better visualize the expression of the two genes within the DEP, we have developed a visualization method, PAGEplot, consisting of three components (Fig. 1): (a) sample-wise expression of the two genes, which is a scatter plot showed the expression levels of the two genes within samples represented by different colors for the two groups, along with the distribution of their expressions (**bottom-left**); (b) summarized expression of the two genes, which used box plots to separately display the summarized expression of individual genes in the two groups (**top-left and bottom-right**); and (c) Fisher’s exact test statistical contingency table, which showed sample frequency counts under each condition (**top-right**). The separation of the two group samples in the plot was indicated by the difference in the relative expression of the two genes. Gene pairs that can distinguish the two phenotypes more distinctly, that is, with stronger discriminative power, are more likely to be determined as DEPs.

### State-of-the-art sepsis diagnostic models

To demonstrate the performance of scGPS2, we compared it to bulk-GPS and scGPS, as well as three published sepsis classification models, including FAIM3/PLAC8 [32], SeptiCyte Lab [33], and sNIP [5]. Similar to scGPS described above, bulk-GPS was directly constructed using the PAGE method, trained by EN on bulk RNA-seq data (GSE189400).

FAIM3/PLAC8 is designed to identify sepsis cases based on gene expression of FAIM3 and PLAC8 using the following formula:


(5) \begin{equation*} {Score}_{FAIM3/ PLAC8}= FAIM3/ PLAC8 \end{equation*}

The SeptiCyte Lab is calculated as:


(6) \begin{equation*} {Score}_{SeptiCyte\ Lab}= PLAC8- PLA2G7+ LAMP1- CEACAM4 \end{equation*}

sNIP score is calculated as:


(7) \begin{equation*} {Score}_{sNIP}=\left( NLRP1- IDNK\right)/ PLAC8 \end{equation*}

The receiver operating characteristic (ROC), precision-recall curves (PRC), the area under the ROC curve (AUROC), and the area under the PRC curve (AUPRC) were used to assess the model performance.

### Functional analysis and visualizations

Gene Ontology biological processes enrichment analysis of the identified gene pairs was performed using Metascape [34] with the default parameters (*P* < .01).

Circos 2D track plot images for genomic data visualization of gene pairs was implemented using RCircos (v1.2.2). Heatmap that shows the relative expression of gene pairs in each cell type was illustrated using a python package PyComplexHeatmap (v1.5.4).

### Experimental validation using FACS

Eight sepsis and eight non-sepsis cases were recruited from patients at department of Critical Care Medicine of the First Affiliated Hospital of Harbin Medical University (Qunli Hospital) as the experimental validation cohort. The patients agreed to donate blood for the current study (2023GS24). 100ul of whole blood was collected from patients with sepsis and non-sepsis for isolating monocytes. The cells were labeled with the following antibodies: CD45 (APC/Cyanine7 anti-human CD45, Biolegend); CD14(PE/Cyanine7 anti-human CD14, Biolegend); HP (Anti-Haptoglobin antibody, Abcam, EPR22856–212); PE secondary antibody (Anti-rabbit IgG(H + L), F(ab’)2 Fragment (PE Conjugate), CST). The samples were incubated with 1× erythrocyte lysis buffer for 7 min. The cells were centrifuged at 500 × g for 5 min and the supernatant was discarded. After washing with PBS, the cells were centrifuged again at 500 × g for 5 min, the supernatant was discarded, and the cells were resuspended in 500ul of BD FACS Flow sheath fluid. Data were collected on a FACSCanto flow cytometer (BD, San Jose, CA). Flow cytometry analysis was performed using FlowJo.

## Results

### Construction of scGPS2 using both single-cell and bulk transcriptome data

Solely based on the single-cell sequencing data, the previous scPAGE algorithm showed strong performance in separating between patients with and without sepsis [13]. In this study, we improved the scPAGE to scPAGE2 algorithm, concentrating on two points (Fig. 1): (1) refined approach of identifying DEP by transforming discrete variables, which merely reflect expression levels, into continuous variables representing relative expression, and (2) fine-tuning the scGPS model using bulk data (see Materials and Methods). After fine-tuning and manually selection, the number of gene pairs reduced from 22 in scGPS to four in scGPS2 using the fine-tuning dataset (GSE189400). The four DEPs were ARG1_CCR7, IRF6_HP, JAM3_PIK3AP1, and TLR9_CLEC4D. The risk score for sepsis was determined as follows:


(8) \begin{equation*} Y=\sum_{i=1}^N\left({Coefficient}_i\ast Relative\_{expression}_{\mathrm{i}}\right) \end{equation*}

**Figure 2:**
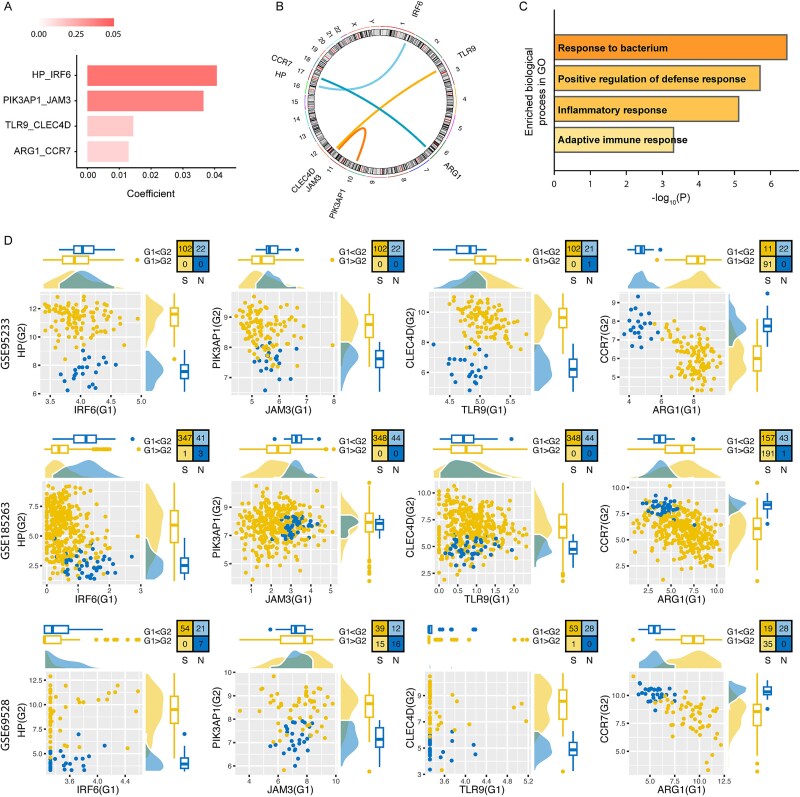
The scGPS2 model developed using the scPAGE2 algorithm. (A) Coefficients of the DEPs in scGPS2. (B) Genomic location of each DEP, with connecting lines representing pairs of DEPs. (C) Enriched biological processes in GO related to the four DEPs. (D) PAGEplot of the DEPs in GSE95233, GSE185263, and GSE69528.

The predicted sepsis score ($Y$) was determined by the weighted sum of the relative expressions of four DEPs, each multiplied by their respective coefficient. The coefficients for ARG1_CCR7, IRF6_HP, JAM3_PIK3AP1, and TLR9_CLEC4D were 0.01439, 0.0129, −0.01868, and − 0.03649, respectively (Fig. 2A, see Materials and Methods).

The genomic location for each gene pair was illustrated in Fig. 2B. The four gene pairs mainly involved in biological processes related to inflammation responses, specifically, response to bacterium, inflammatory response, and adaptive immune response (Fig. 2C), indicating the close association with sepsis. The validation of scGPS2 was conducted across eight datasets spanning three platforms. The expression patterns of the gene pairs remained consistent across multiple datasets (platform representatives in Fig. 2D and other datasets in Supplementary Fig. S1). For instance, in sepsis samples, high expression of *HP* and low expression of *IRF6* were consistently observed, whereas in normal samples, the pattern was opposite with low HP expression and high *IRF6* expression across all datasets. Conversely, sepsis samples consistently exhibited high *ARG1* expression and low *CCR7* expression, while normal samples consistently displayed high *CCR7* expression and low *ARG1* expression across the various datasets.

### scGPS2 demonstrates high cross-platform generalizability

**Figure 3:**
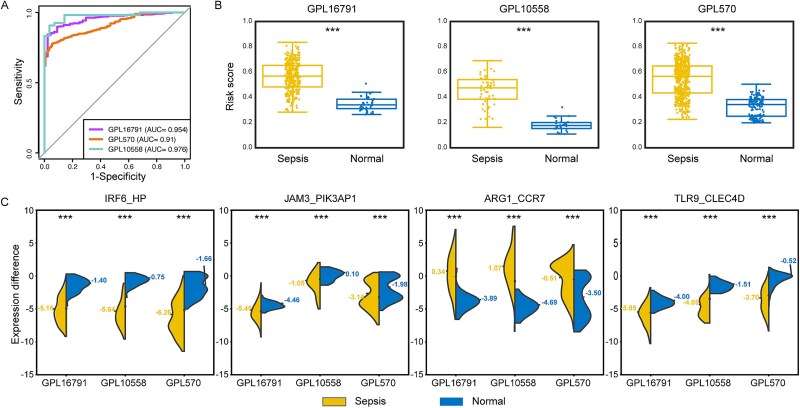
Performance evaluation of scGPS2. (A) ROC curves demonstrating sensitivity and specificity of scGPS2 on each platform. (B) Risk score generated by scGPS2 for each sample in every platform. (C) Distribution of expression differences for each DEP across platforms. The differences were calculated according to the order of the genes in DEP names. The mean value of expression difference in each group and platform was marked. ‘***’ represented *P* < .001.

We used the ROC, PRC, and the corresponding area under the curve to evaluate the model performance and cross-platform generalizability of scGPS2 on each platform. scGPS2 achieves a robust and consistent performance across three platforms (Fig. 3A), including NGS (AUROC = 0.954 in GPL16791) and microarray platforms (AUROC = 0.91 in GPL570 and AUROC = 0.976 in GPL10558). Furthermore, the risk scores exhibited a significant distinction between sepsis and normal samples on each platform (Fig. 3B; Student’s T-Test; GPL16791, P-value = 5.24e-41; GPL570, P-value = 1.88e-90; GPL10558, P-value = 5.47e-24). A higher risk score indicated an increased risk of developing sepsis, whereas a lower risk score suggested a higher possibility of the sample being normal. ﻿The disparity in gene expression between the four DEPs exhibited a consistent opposing trend across various platforms in both sepsis and normal samples (Fig. 3C). For instance, the expression variance between IRF6 and HP was consistently lower in sepsis samples compared to normal samples across all three platforms. Conversely, the difference in expression levels between *ARG1* and *CCR7* was consistently higher in sepsis samples compared to normal samples. Taken together, scGPS2 demonstrated robust and consistent performance in distinguishing between sepsis and normal samples across diverse platforms. This suggests that the inflammation-related response model, scGPS2, holds promise as an effective predictor for identifying sepsis samples, consistent with the notion that sepsis is fundamentally an inflammatory disease.

### scGPS2 outperforms state-of-the-art models

**Figure 4:**
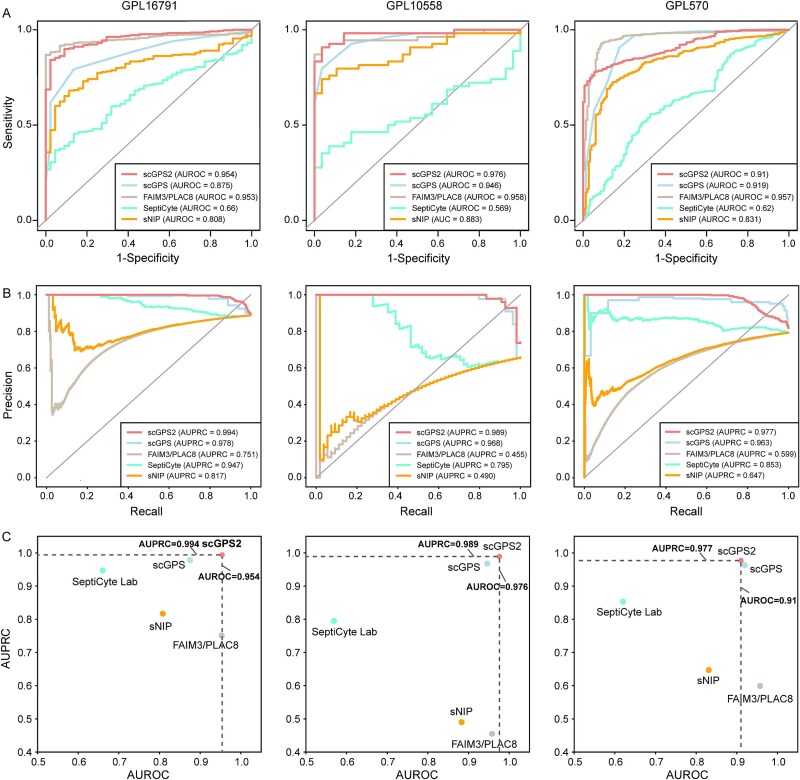
Performance comparison of scGPS2 and state-of-the-art classification models. (A) ROC curves illustrating sensitivity and specificity of scGPS2 in comparison to scGPS, FAIM3/PLAC8, SeptiCyte lab, and sNIP across three platforms. (B) PRC illustrating precision and recall of scGPS2 in comparison to scGPS, FAIM3/PLAC8, SeptiCyte lab, and sNIP across three platforms. (C) Scatter plot for model AUPRC and AUROC for each platform.

To assess the efficacy of scGPS2, we conducted a comparative analysis of its performance against four state-of-the-art sepsis classification models: scGPS, FAIM3/PLAC8, SeptiCyte Lab, and sNIP, as detailed in the Materials and Methods. Our findings demonstrated that scGPS2 exhibited consistently superior performance across platforms (Fig. 4). For the NGS platform GPL16791, scGPS2 showed the highest AUROC (AUROC = 0.954; Fig. 4A), surpassing the performance of the other four models (AUROC = 0.875 for scGPS, AUROC = 0.953 for FAIM3/PLAC8, AUROC = 0.66 for SeptiCyte Lab, and AUROC = 0.808 for sNIP). On the other hand, for one of the microarray platforms, GPL10558, scGPS2 showcased exceptional performance with an AUROC of 0.976, outperforming the other classifiers: AUROC of 0.946 for scGPS, AUROC of 0.958 for FAIM3/PLAC8, AUROC of 0.569 for SeptiCyte Lab, and AUROC of 0.883 for sNIP. For GPL570, while scGPS2 still demonstrated a relatively strong classification performance with an AUROC of 0.91, it was surpassed by FAIM3/PLAC8 (AUROC = 0.957) and scGPS (AUROC = 0.919), ranking third in performance. Nevertheless, scGPS2 showed its strength in terms of the trade-off between the true positive rate and the positive predictive value, which is the AUPRC (Fig. 4B). scGPS2 achieved values of 0.994 for GPL16791, 0.989 for GPL10558, and 0.977 for GPL570, securing the top position ahead of other methods, while scGPS remained in the second position. Taken into consideration both AUROC and AUPRC, scGPS2 showcased commendable classification performance across all of the testing platforms (Fig. 4C), implying its potential for effectively diagnosing sepsis.

### Distinctive roles of DEP genes: Dominant and reference

**Figure 5:**
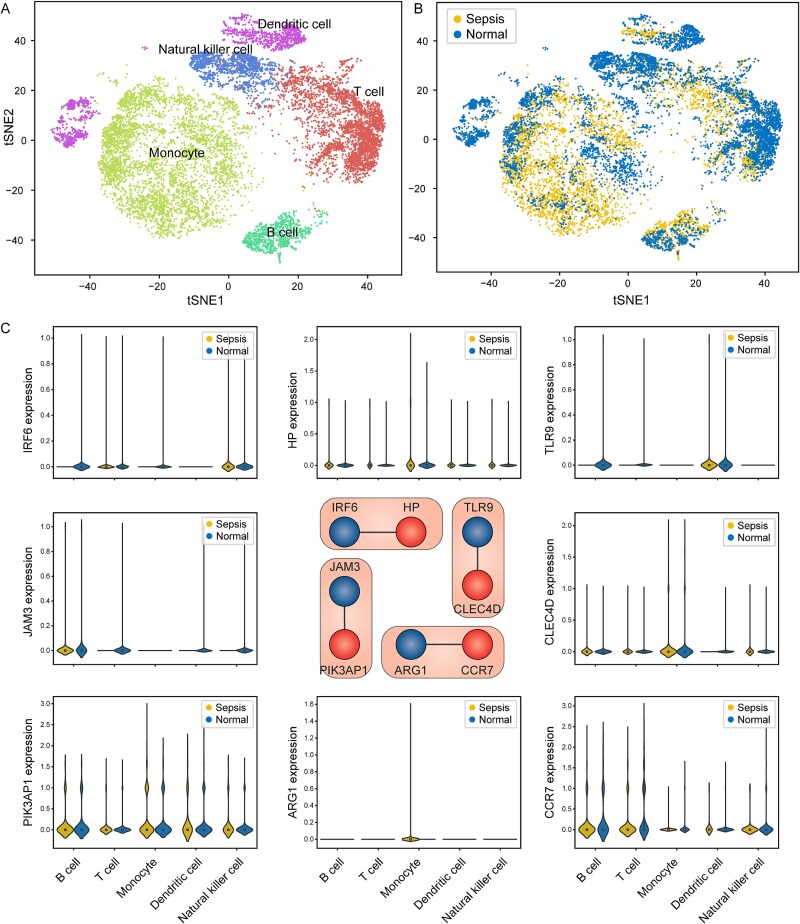
Gene expression in the four DEPs across cell types in single-cell dataset. (A) Cell clustering using t-SNE with annotations for B cells, dendritic cells, monocytes, NK cells, and T cells. (B) Cell clustering using t-SNE with annotations for sepsis and normal samples. (C) the central panel displays the DEPs. IRF6, TLR9, JAM3, and ARG1 were reference genes, while HP, CLEC4D, PIK3AP1, and CCR7 iwere dominant genes. The surrounding panel demonstrates the expression of each gene across cell types.

Since the original scGPS was constructed using gene expression data from single-cell datasets, we hypothesized that the efficacy of scGPS2 might stem from its contribution at the individual cell type level. Upon mapping the high-dimensional points to two dimensions using t-distributed stochastic neighbor embedding (t-SNE) based on gene expression within each cell, the resulting visualization exhibited distinct and well-clustered cell types corresponding to annotations, namely B cell, DC, monocyte, NK, and T cell (Fig. 5A). No apparent differentiation was observed between sepsis and normal cells (Fig. 5B). Next, we proceeded to examine the expression of genes within each of the four DEPs across cell types (Fig. 5C). We noticed that within each DEP, two genes displayed distinct expression patterns, wherein one assumed a dominant role while the other served as a reference. The dominant gene, defined as expressed fluctuatingly across various cell types, included *HP*, *CLEC4D*, *PIK3AP1*, and *CCR7*. The reference gene, defined as consistent low expression across cell types, included *IRF6*, *TLR9*, *JAM3*, and *ARG1*. For instance, *CCR7* showed elevated expression in T cells, B cells and NK cells, whereas its counterpart gene, *ARG1*, consistently displayed low expression across all five cell types. Taken together, the scPAGE2 algorithm identified sepsis-specific genes that are overlooked by traditional biomarker identification methods, regardless of the expression level in individual gene.

### Cell type specificity of DEPs in sepsis

**Figure 6:**
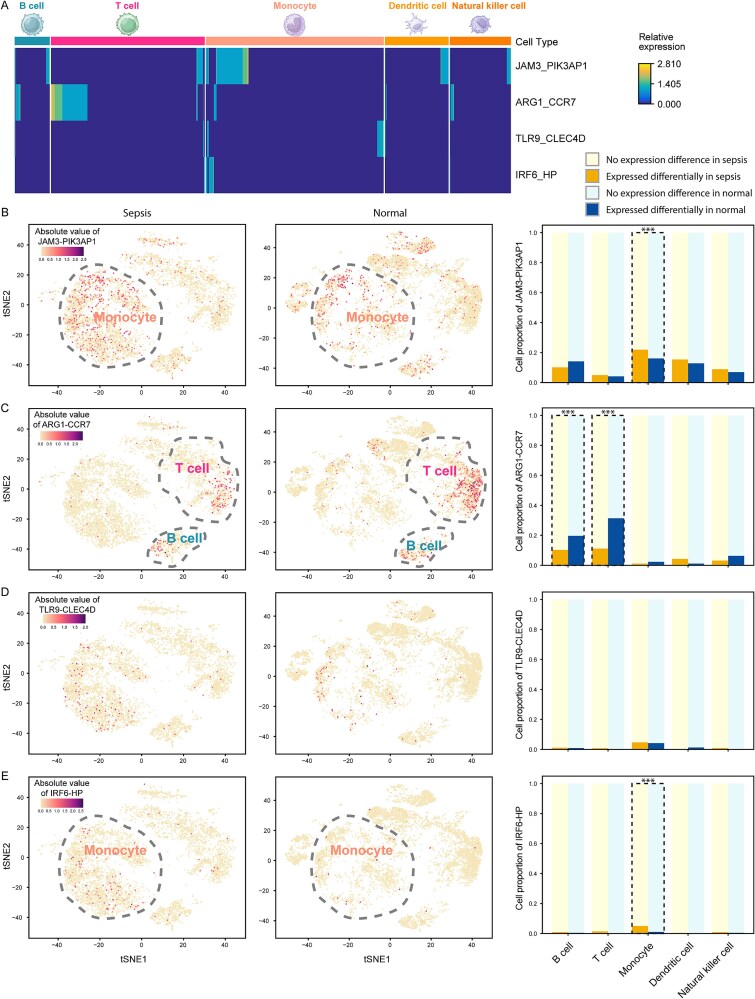
Roles of DEPs in specific cell types. (A) Heatmap presenting the relative expression of the DEPs across various cell types. (B-E) the left two panels displayed cell clustering, with color representing the absolute value of the relative expression of JAM3-PIK3AP1, ARG1-CCR7, TLR9-CLEC4D, and IRF6-HP in sepsis and normal samples, respectively. The right panel illustrates the cell proportion for each DEP based on its relative expression. “***” represented *P* < .001.

After scrutinizing individual gene expressions in single-cell samples, we questioned whether the relative expression of each DEP could accurately represent a distinct cell type. Notably, we observed a significant elevation in the relative expression of ARG1-CCR7 in both B cells and T cells. Conversely, TLR9-CLEC4D and IRF6-HP exhibited specific enrichment primarily in monocytes. Interestingly, JAM3-PIK3AP1 emerged as a universal marker, displaying high relative expression across all cell types (Fig. 6A).

We then investigated whether the cell type-specific expression of these DEPs is associated with sepsis. Despite the globally high relative expression of JAM3-PIK3AP1 across various cell types, the proportion of cells with the high relative expression was significantly higher in sepsis samples compared to normal samples in monocytes (Fig. 6B; Fisher’s exact test, P-value = 8.15e-07). The B cell and T cell marker DEP, ARG1-CCR7 exhibited a notable decrease in the proportion of ARG1-CCR7-enriched cells in sepsis compared to normal samples (Fig. 6C; Fisher’s exact test, P-value = 6.31e-05 in B cell and P-value = 5.25e-49 in T cell). This aligns with previous findings suggesting that CCR7 deficiency can exacerbate skin inflammation [35], reinforcing ARG1-CCR7’s role as a B and T cell marker in sepsis.

**Figure 7:**
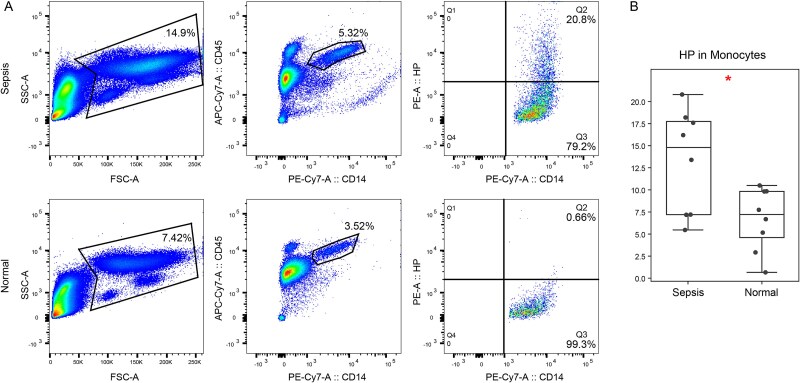
Experimental validation of HP expression in monocyte using FACS. (A) Representative images of haptoglobin expression in blood monocytes from patients with and without sepsis. (B) Boxplot shows the cell proportion of HP-positive monocyte in each sample. ‘*’ represented *P* < .05.

Among the two identified monocyte maker DEPs, only IRF6-HP showed a significantly higher proportion of cells in sepsis samples compared to normal samples (Fig. 6D-E; Fisher’s exact test, P-value = 2.18e-14). To assess the diagnostic value of HP, which encodes haptoglobin, we further performed an experimental validation of haptoglobin expression in monocytes using FACS in an independent cohort (Fig. 7A; see Materials and Methods). The results demonstrated a significant increase in the expression of HP in sepsis patients compared to normal samples (Fig. 7B; Student’s T-Test, P-value = 1.97e-02). In sepsis, the body’s triggered inflammatory response against the underlying infection leads to elevated haptoglobin levels as part of the acute-phase reactants [36]. Haptoglobin plays a crucial role in scavenging free hemoglobin released from damaged red blood cells, thus mitigating oxidative damage and supporting immune function [37].

Taken together, the four DEPs involved in scGPS2 specifically serve their cell type-related roles in the context of sepsis: the inhibition of ARG1-CCR7 in sepsis B cells and T cells, while the activation of JAM3-PIK3AP1 and IRF6-HP in sepsis monocytes.

### Generalization of scPAGE2 in AML

To illustrate the effectiveness of scPAGE2 and ensure a more direct comparison with scPAGE, we expanded its application to AML, the disease for which scPAGE was initially developed. Following the same procedure used to identify the sepsis scGPS2, we first applied scPAGE to single-cell data from AML and normal mouse bone marrow, resulting in the AML scGPS model, which included 30 DEPs. After fine-tuning with the bulk dataset (GSE74690), the AML scGPS2 model was refined to include 13 DEPs. The risk score for AML scGPS2 was determined as outlined in Equation 4. The DEPs and their corresponding coefficients are detailed in Supplementary Table S2.

Although the number of DEPs decreased from 30 in AML scGPS to 13 in AML scGPS2, the classification performance improved and remained stable across five validation datasets (Supplementary Fig. S2). The average AUC across the five validation cohorts reached 0.995, compared to 0.956 for AML scGPS. Both GSE84988 and GSE121122 achieved identical AUC scores (AUC = 1) for AML scGPS and AML scGPS2. Notably, improvements were observed in GSE105049 (scGPS2: AUC = 1; scGPS: AUC = 0.83) and GSE78691 (scGPS2: AUC = 1; scGPS: AUC = 0.95). The application of scPAGE2 in AML highlights its generalization capability across multiple diseases.

## Discussion

Sepsis occurs when an infection initiates a life-threatening chain reaction throughout the body [38, 39]. Early diagnosis of sepsis presents challenges due to its manifestation in vastly different ways [40]. Omics technologies are powerful tools for constructing models to diagnose diseases [41]. While state-of-the-art models have been developed, their poor generalization has not yet led to an improved diagnostic ability for sepsis. The PAGE algorithm was created to circumvent inter-sample comparisons and enhance model generalization using relative expression within a single sample [12–17]. Building upon our previously developed scPAGE algorithm, we have devised a scGPS model that effectively classifies sepsis samples [13].

This study introduces an enhanced sepsis classification model, scGPS2, refining our previous scGPS model. Improvements of scGPS2 include refining DEP construction into continuous variables and fine-tuning the scGPS model using bulk data. scGPS2, which involved ARG1-CCR7, IRF6-HP, JAM3-PIK3AP1, and TLR9-CLEC4D, demonstrated superior performance in distinguishing sepsis from normal samples across NGS and microarray platforms. scGPS2 surpasses state-of-the-art models, namely scGPS, FAIM3/PLAC8, SeptiCyte Lab, and sNIP, with an average AUROC of 0.947 and an average AUPRC of 0.987. In addition, we developed a visualization method, PAGEplot, to dedicatedly illustrate and compare the expression pattern between two genes, *i.e.*, DEPs generated from PAGE. PAGEplot showed sample-wise and summarized expression of the two genes, as well as the statistical results of the DEP, providing an intuitive presentation for pairwise visualization.

The superior performance of scPAGE2 stems from its integration of a wealth of information in single-cell transcriptome data, the transfer learning capability to apply knowledge from one domain to another, and the strong generalization ability of PAGE. Single-cell sequencing provides a higher resolution, allowing for the analysis of individual cells within a heterogeneous population [42]. Moreover, with enabling the identification of cell-to-cell variability, it uncovers differences in gene expression and cell states [43]. In the context of sepsis, the gene expression profiles of individual immune cells are instrumental in identifying the specific cell types that are activated or suppressed during the course of the disease in comparison to the traditional bulk analysis. This detailed analysis is crucial for understanding the cellular dynamics that contribute to immune paralysis. Single Cell Analysis: Technologies like single-cell RNA sequencing allow researchers to study the gene expression profiles of individual immune cells. This helps identify specific cell types that are activated or suppressed during sepsis. In sepsis, there is often a hyper-inflammatory response followed by immune suppression. Single-cell studies can reveal which specific cells are driving inflammation or contributing to immune paralysis.

Transfer learning leverages knowledge gained from one domain and apply it to another, typically when the amount of data available for the target task is limited. By using pre-trained models, transfer learning can significantly reduce the requirement of labeled data needed for training. It allows models to benefit from the general features and patterns learned from one task and adapt them to improve performance on a new, related task [44]. PAGE utilized the relative expression within one sample to avoid the various expression scales across samples. This leads to the strong generalization ability of PAGE. Therefore, PAGE can effectively extract information from single-cell sequencing data and enhance efficiency through transfer learning.

As expected, the four DEPs screened from immune-related genes exhibited a strong association with inflammation response-related pathways [45, 46]. The classification capability of immune-related genes in sepsis emphasizes the dynamic changes in immune regulation within the body when subjected to sepsis. Moreover, we performed a comprehensive analysis of the four DEPs within scGPS2 on single cell level. The relative expression of the DEPs across various cell types demonstrated distinct expression patterns specific to certain cell types. However, these patterns alone are insufficient for accurately distinguishing among different cell types, indicating that reliance on a single type of cell is inadequate for diagnosing sepsis. Nonetheless, the pre-training step in single-cell data offers valuable insights into potential variations of DEPs in functional roles across diverse cell types, enhancing the overall understanding of the immune response in sepsis. Besides, these DEPs exhibited significant differences in expression between sepsis and normal samples within their associated cell types.

Notably, IRF6-HP and JAM3-PIK3AP1 showed a significant increase in monocyte cell proportion during sepsis compared to normal samples. Haptoglobin, an acute-phase protein encoded by the HP gene, is primarily synthesized by the liver and released into the bloodstream in response to inflammation, infection, or tissue damage [47]. The elevation of HP in monocytes during sepsis showed its potential as a biomarker in indicating inflammatory response against the underlying infection [37, 38]. Therefore, elevated expression of HP in monocytes among patients may indicate an increased likelihood of sepsis in clinical settings. PIK3AP1, which encodes the BCAP protein, displayed widespread expression across various cell types but exhibited differential expression only in monocytes between sepsis and normal individuals. The elevated expression in monocytes in sepsis suggests its potential to differentiate sepsis from non-sepsis, which should be assessed in the experimental validation cohorts before considering as a novel sepsis diagnostic marker.

Conversely, ARG1-CCR7 demonstrated a substantial decrease in the proportions of B and T cells during sepsis compared to normal samples. Typically, CCR7 is a chemokine receptor associated with the migration and homing of B and T cells within the immune system [48]. During sepsis, characterized by a dysregulated immune response to infection, alterations in the expression of CCR7 [35] may be attributed to changes occurring within these cells.

The generated model using scPAGE2, which relies solely on the gene expression of four gene pairs, enables a simple clinical implementation of sepsis diagnosis in routine diagnostic workflows. After collecting blood samples from patients, quantitative reverse transcription polymerase chain reaction (qRT-PCR) should be utilized to detect the gene expression levels of the model genes. In compliance with data privacy regulations, these gene expression levels can then be input into the model using standard computational resources. Due to its expedited processing time, a rapid turnaround for diagnostics is anticipated, thereby enhancing decision-making in critical care settings.

However, this study has some limitations. Diverse patient populations—including additional platforms, specific age groups, and patients with various comorbidities and symptoms—are crucial factors influencing the model’s performance and generalizability. Therefore, we advocate for additional related studies and the publication of transcriptomic data. This will enable us to incorporate the new information to adjust for confounding factors, thereby enhancing the model’s confidence and robustness. Furthermore, sepsis presents with varied symptoms, and patients exhibiting the same symptoms are not always experiencing sepsis. Therefore, identification models that can distinguish between patients with similar symptoms are crucial for clinical use. Future work will focus on comparisons between sepsis patients with diverse symptoms and non-sepsis patients displaying the same symptoms. In addition, although scGPS2 was developed from single-cell transcriptome data and validated in cell types, it reckoned without cell type information during the model training phase. Future model improvements will involve the consideration on cell type information when train the models.

## Conclusions

In conclusion, the development of scGPS2 based on scPAGE2 algorithm, by enhancing classification effectiveness and deciphering key marker DEPs across different cell types, helps in advancing the progress of sepsis adjunctive diagnostics.

**Key Points**

- scPAGE2 is a PAGE-based sepsis diagnostic model that seamlessly transferred cell-layer information to bulk transcriptome.
- scPAGE2 demonstrates superior performance over existing models across multiple platforms.
- The robustness of scPAGE2 was further validated by in vitro experiment of the IRF6-HP gene pair in monocytes.

## List of abbreviations

AUC, Area under the curve; DC, Dendritic cell; DEPs, Differentially expressed gene pairs; EN, Elastic net; FACS, Fluorescence-activated cell sorting; NGS, Next-generation sequencing; NK, Natural killer; PBMC, Peripheral blood mononuclear cell; lncRNAs, Long non-coding RNAs; PRC, Precision-recall curves; ROC, Receiver operating characteristic; scGPS, Single-cell gene pair signatures; scPAGE, Single-cell pair-wise analysis of gene expression; scRNA, Single-cell RNA; SFS, Sequential forward selection; TPM, Transcript per million.

## Supplementary Material

Supplementary_Material_bbae661

## Data Availability

The datasets used and/or analyzed during the current study are available from the corresponding author on reasonable request.
