# Supplementary material for: PAGE-based transfer learning from single-cell to bulk sequencing enhances model generalization for sepsis diagnosis
Source: Brief Bioinform. 2024 Nov 22;26(1):bbae661. doi: 10.1093/bib/bbae661 (PMC11962595; doi:10.1093/bib/bbae661)
Supplement: Supplementary_Material_bbae661 [file supplementary_material_bbae661.pdf]

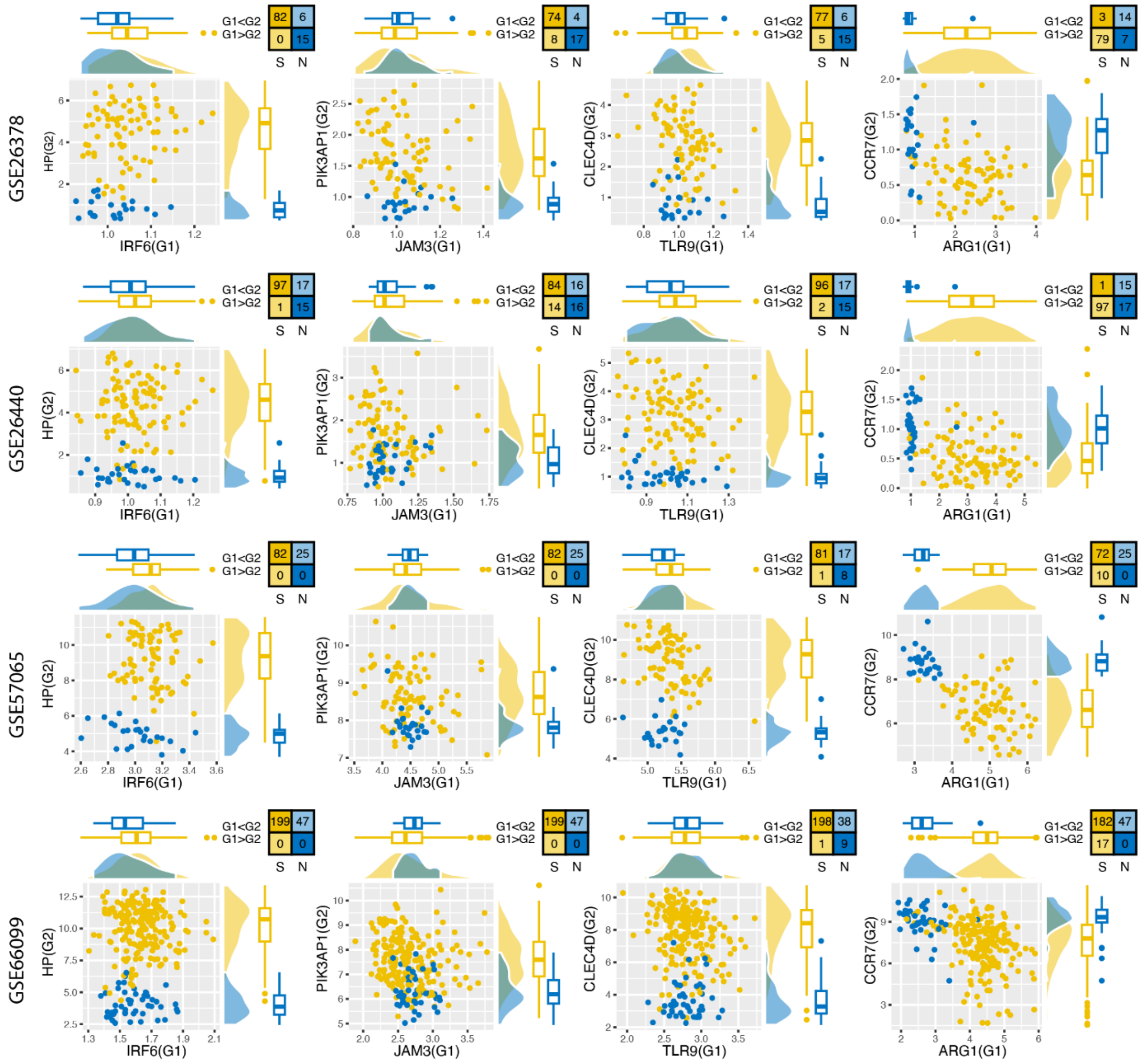

Supplementary Figure S1. Scatter plot displaying the normalized expression of the DEPs in each sample of the datasets. Yellow points represent sepsis samples, while blue points represent normal samples. The contingency table in the top right corner of the scatter plot shows the sample count for each condition.

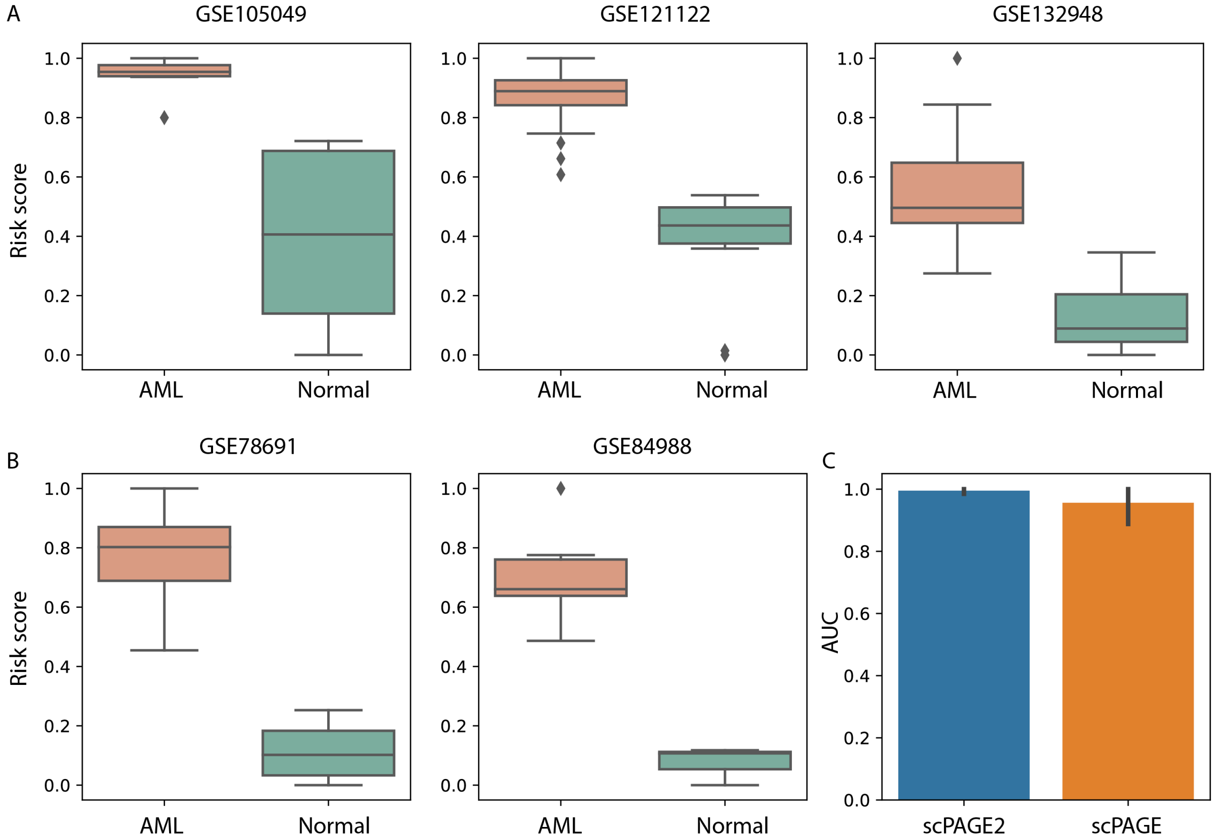

Supplementary Figure S2. (A-B) Box plots showing the risk score predicted by scPAGE2 in AML datasets. (C) The comparison between AUC of scPAGE2 and scPAGE.

Supplementary Table S1. Detailed information of the DEPs.

DEP	Entreze ID 1	Gene Symbol 1	Gene Description 1	Entreze ID 2	Gene Symbol 2	Gene Description 2
DUSP1_TLR9	1843	DUSP1	dual specificity phosphatase 1	54106	TLR9	toll like receptor 9
TLR9_S100A12	54106	TLR9	toll like receptor 9	6283	S100A12	S100 calcium binding protein A12
TLR9_MAFB	54106	TLR9	toll like receptor 9	9935	MAFB	MAF bZIP transcription factor B
MUC1_HP	4582	MUC1	mucin 1, cell surface associated	3240	HP	haptoglobin
TLR9_GAB1	54106	TLR9	toll like receptor 9	2549	GAB1	GRB2 associated binding protein 1
CAV1_JAM3	857	CAV1	caveolin 1	83700	JAM3	junctional adhesion molecule 3
PGLYRP2_TLR9	114770	PGLYRP2	peptidoglycan recognition protein 2	54106	TLR9	toll like receptor 9
PPARG_PROCR	5468	PPARG	peroxisome proliferator activated receptor gamma	10544	PROCR	protein C receptor
IRF6_HP	3664	IRF6	interferon regulatory factor 6	3240	HP	haptoglobin
ARG1_CLEC4C	383	ARG1	arginase 1	170482	CLEC4C	C-type lectin domain family 4 member C
PPARG_IL18R1	5468	PPARG	peroxisome proliferator activated receptor gamma	8809	IL18R1	interleukin 18 receptor 1
ARG1_PROCR	383	ARG1	arginase 1	10544	PROCR	protein C receptor
ARG1_IL18R1	383	ARG1	arginase 1	8809	IL18R1	interleukin 18 receptor 1
ARG1_CXCR3	383	ARG1	arginase 1	2833	CXCR3	C-X-C motif chemokine receptor 3
ARG1_CCR7	383	ARG1	arginase 1	1236	CCR7	C-C motif chemokine receptor 7
LGMN_ARG1	5641	LGMN	legumain	383	ARG1	arginase 1
PPARG_CXCR3	5468	PPARG	peroxisome proliferator activated receptor gamma	2833	CXCR3	C-X-C motif chemokine receptor 3
TLR9_CLEC4D	54106	TLR9	toll like receptor 9	338339	CLEC4D	C-type lectin domain family 4 member D
TLR9_HP	54106	TLR9	toll like receptor 9	3240	HP	haptoglobin
TLR2_TLR9	7097	TLR2	toll like receptor 2	54106	TLR9	toll like receptor 9
JAM3_PIK3AP1	83700	JAM3	junctional adhesion molecule 3	118788	PIK3AP1	phosphoinositide-3-kinase adaptor protein 1
TLR9_EGR1	54106	TLR9	toll like receptor 9	1958	EGR1	early growth response 1

Supplementary Table S2. DEPs and coefficients in AML scGPS2.

DEP	Coefficient
Cd8a-Cd28	-0.0524
Ell3-Egln3	0.04
Gldc-Blk	0.1129
Gli1-Bcl11a	0.0947
Gli1-Cxcr2	0.0034
Havcr2-Gfi1	0.0182
Ildr1-Cdkn1a	-0.0344
Il1a-Gstt2	0.052
Klrg1-Hoxa9	-0.0603
Klrg1-Ifngr1	-0.0094
Ptcra-Pax5	0.003
Shh-Capn5	0.0361
and Vtn-Irf4	-0.0521
